# Determinants of the Over-Anticoagulation Response during Warfarin Initiation Therapy in Asian Patients Based on Population Pharmacokinetic-Pharmacodynamic Analyses

**DOI:** 10.1371/journal.pone.0105891

**Published:** 2014-08-22

**Authors:** Minami Ohara, Harumi Takahashi, Ming Ta Michael Lee, Ming-Shien Wen, Tsong-Hai Lee, Hui-Ping Chuang, Chen-Hui Luo, Aki Arima, Akiko Onozuka, Rui Nagai, Mari Shiomi, Kiyoshi Mihara, Takashi Morita, Yuan-Tsong Chen

**Affiliations:** 1 Department of Biopharmaceutics, Meiji Pharmaceutical University, Tokyo, Japan; 2 Laboratory for International Alliance on Genomic Research, RIKEN Center for Integrative Medical Sciences, Kanagawa, Japan; 3 Departments of Medicine and Neurology, Chang Gung Memorial Hospital and Chang Gung University College of Medicine, Taoyuan, Taiwan; 4 Institute of Biomedical Sciences, Academia Sinica, Taipei, Taiwan; 5 Faculty of Pharmacy, Center for Clinical Pharmacy, Musashino University, Tokyo, Japan; 6 School of Medicine, Juntendo University, Tokyo, Japan; Maastricht University Medical Center, Netherlands

## Abstract

To clarify pharmacokinetic-pharmacodynamic (PK-PD) factors associated with the over-anticoagulation response in Asians during warfarin induction therapy, population PK-PD analyses were conducted in an attempt to predict the time-courses of the plasma *S*-warfarin concentration, Cp(S), and coagulation and anti-coagulation (INR) responses. In 99 Chinese patients we analyzed the relationships between dose and Cp(S) to estimate the clearance of *S*-warfarin, CL(S), and that between Cp(S) and the normal prothrombin concentration (NPT) as a coagulation marker for estimation of IC_50_. We also analyzed the non-linear relationship between NPT inhibition and the increase in INR to derive the non-linear index λ. Population analyses accurately predicted the time-courses of Cp(S), NPT and INR. Multivariate analysis showed that CYP2C9*3 mutation and body surface area were predictors of CL(S), that *VKORC1* and *CYP4F2* polymorphisms were predictors of IC_50_, and that baseline NPT was a predictor of λ. CL(S) and λ were significantly lower in patients with INR≥4 than in those with INR<4 (190 mL/h vs 265 mL/h, P<0.01 and 3.2 vs 3.7, P<0.01, respectively). Finally, logistic regression analysis revealed that CL(S), ALT and hypertension contributed significantly to INR≥4. All these results indicate that factors associated with the reduced metabolic activity of warfarin represented by CL(S), might be critical determinants of the over-anticoagulation response during warfarin initiation in Asians.

**Trial Registration:**

ClinicalTrials.gov NCT02065388

## Introduction

Initiation therapy with warfarin has been hampered by two major problems, one of which is a large inter-individual variability in the maintenance dose and the other is an over-anticoagulation response leading to bleeding complications, especially before establishment of the maintenance dose. Genetic polymorphisms of *VKORC1* and *CYP2C9* have been established as major determinants of inter-individual variability in the maintenance dose, especially in whites. [1], [2] Accordingly, pharmacogenetic-based algorithms or a table and guidelines for estimating the initial dose of warfarin necessary for achieving a therapeutic International Normalized Ratio (INR) are currently available [3], [4], [5], [6].

With regard to bleeding complications, although many clinical prediction scores/schemes including an age of >60–75 yr, labile INR, concomitant drugs, abnormal kidney/liver function and several complications, e.g., hypertension, have been reported, [7], [8], [9] validation studies have exhibited their insufficient predictive accuracies for routine use in practice. [10], [11] Up to now, none of these bleeding prediction studies have considered the pharmacokinetic-pharmacodynamic (PK-PD) related risk factors of warfarin for over- anticoagulation, except for one study [7] in which *CYP2C9* mutation related to the low clearance of *S*-warfarin was included as a risk factor.

As the risk of bleeding complications as well as the antithrombotic effects of warfarin therapy are related to INR management, [12], [13] recent two large prospective trials have evaluated the impact of genotype-based warfarin dosing on INR control in terms of the time within the therapeutic range during initiation therapy. [14], [15] However, the results of these two studies were completely different. The EU-PACT study showed that genotype information clearly improved anti-coagulation control and the rate of INR≥4. [14] On the other hand, the COAG study found no improvement in either anti-coagulation control or the rate of INR≥4. [15] These trials have also shown that almost 20–30% of patients have experienced an over-anticoagulation response during induction therapy even after consideration of the two genotypes. [14], [15] In addition to these observations, a previous study has reported that information on both genotypes, the early INR response and patients’ characteristics accounted for only 16% of inter-patient variability in the time required to reach INR>4, [16] indicating the involvement of currently unknown factors in the over-anticoagulation response to warfarin.

Furthermore, previous studies have reported the association of either of the variants of *CYP2C9* and *VKORC1*, or their combination, with the risk of hemorrhage or an over-anticoagulation response in whites, but not in African Americans, [17], [18] indicating that the predictability of risk for over-anticoagulation might be inconsistent among different races. Since Asians represent a highly homogeneous population with regard to the two genes (more than 80% of Asians possesses a combination of CYP2C9*1/*1 and VKORC1*2/*2), we hypothesized that the contribution of genotypes to over-anticoagulation response would be different in Asians from that in the more heterogeneous white population.

Based on these previous observations, we considered that predictions of the time-course of the INR and contributors related to the PK-PD relationship after warfarin initiation would be of great clinical value for revealing factors responsible for the over-anticoagulation response, and accordingly we decided to employ a mechanism-based PK-PD approach using a population pharmacokinetic technique. The goal of the present study was to clarify which parameter(s) of PK-PD of warfarin, including their predictors such as *CYP2C9* and *VKORC1* polymorphisms, contributes to the over-anticoagulation response during the induction therapy in Asian patients.

## Methods

### Patients

**Figure 1:**
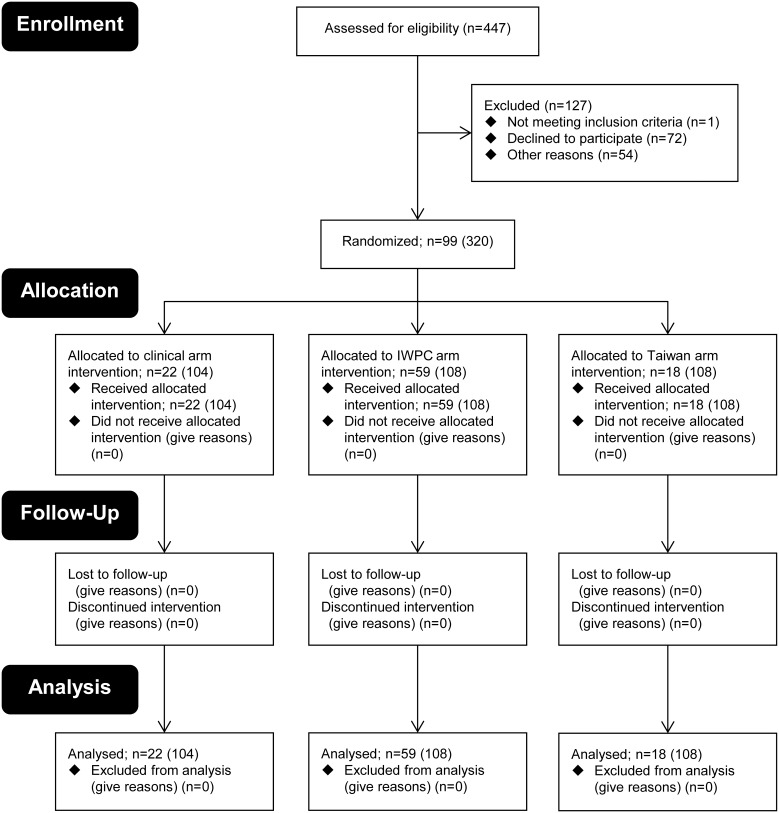
Flow diagram of the randomized trial of the control and genotype groups. The patient numbers participated during the entire date range of the study were shown in parentheses and samples analyzed in this study were collected from 2010 to 2012.

The present study (n = 99) represents part of a randomized prospective trial to compare genotype-guided (n = 77) [3], [4], [19] vs. standard warfarin dosing (n = 22) [20] conducted at outpatient clinics in Taiwan (Figure 1). [21] The entire date range for participant recruitment and follow-up was September 1, 2009–December 31, 2013 and samples analyzed in this study were collected from July 1, 2010 to February 1, 2012. Each patient received at least three fixed initial loading doses (1.5 times the predicted maintenance dose) of warfarin based on their *CYP2C9* and *VKORC1* genotypes by using either a dosing table (n = 18) [19] or dosing equations (n = 59), [3], [4] or on the standard warfarin initiation protocol without reference to genetic information (n = 22). [20] A subsequent dosing titration was performed based on the INR in order to achieve target INR values of 2.0–3.0. The maintenance dose in this study was defined as the daily dose given to patients when two consecutive INR values measured at least one week apart were found to be controlled within the therapeutic range of 2 to 3, with no dose adjustments. Three bleeding complications (GI 1 case; eye 2 cases) occurred during the follow-up period. Demographic characteristics of patients were shown in Table 1. Concurrent medications that might have affected warfarin metabolism included amiodarone (n = 6), rosuvastatin (n = 3), diltiazem (n = 16), and phenytoin (n = 2).

**Table 1:** Patient demographics.

Variable^a^	n = 99	INR<4 (n = 64)	INR≥4 (n = 35)	P-value^d^
Age (yr)	64.5±15.2	60.7±15.9	71.4±10.8	0.002
Gender (F/M)	39/60	25/39	14/21	0.547
Body weight (kg)	68.4±12.4	69.3±12.4	66.7±12.5	0.279
Height (cm)	163.6±8.2	164.6±7.7	161.9±8.9	0.104
Body surface area (m^2^)	1.74±0.18	1.75±0.17	1.71±0.18	0.111
Algorithm (Genotype/Standard)	77/22 (77.8%)	55/9 (85.9%)	22/13 (62.9%)	0.009
Starting dose (mg/day)	4.34±0.98	4.37±0.99	4.28±0.96	0.709
Maintenance dose (mg/day)^b^	2.94±1.35	3.26±1.31	2.31±1.20	0.000
NPT_0_ (µg/mL)	118.2±22.1	119.0±24.4	116.6±17.4	0.777
INR_0_	1.05±0.10	1.05±0.11	1.04±0.08	0.447
INR^c^	2.25±0.88	2.10±0.62	2.52±1.16	0.000
AST (IU/L)	29.2±16.3	28.9±16.7	29.8±15.7	0.471
ALT (IU/L)	27.2±17.5	23.9±15.1	33.3±20.2	0.011
Creatinine clearance (mL/min)	73.3±36.6	80.0±37.2	61.1±32.6	0.009
Alcohol (+/−)	28/71 (28.3%)	23/41 (35.9%)	5/30 (14.3%)	0.018
Smoking (+/−)	20/79 (20.2%)	15/49 (23.4%)	5/30 (14.3%)	0.207
**Complications**				
Diabetes mellitus (+/−)	19/80 (19.2%)	11/53 (17.2%)	8/27 (22.9%)	0.333
Hypertension (+/−)	66/33 (66.7%)	36/28 (56.3%)	30/5 (85.7%)	0.002
Hepatic disease (+/−)	10/89 (10.1%)	8/56 (12.5%)	2/33 (5.7%)	0.241
Chronic kidney disease (+/−)	16/83 (16.2%)	5/59 (7.8%)	11/24 (31.4%)	0.003
**Indication**				
Atrial fibrillation	54 (54.5%)	27 (42.2%)	27 (77.1%)	0.001
Stroke	29 (29.3%)	16 (25.0%)	13 (37.1%)	0.150
Deep vein thrombosis	25 (25.3%)	22 (34.4%)	3 (8.6%)	0.003
Pulmonary embolism	8 (8.1%)	6 (9.4%)	2 (5.7%)	0.413
Coronary artery embolism	1 (1.0%)	1 (1.6%)	0 (0.0%)	0.646
Others	14 (14.1%)	9 (14.1%)	5 (14.3%)	0.598
**Genotypes Wild/Hetero/Homo (MAF)**				
CYP2C9*3 rs1057910 (1075 A>C)	88/11/0 (0.056)	59/5/0 (0.039)	29/6/0 (0.086)	0.141
VKORC1*2 rs9923231 (–1639 G>A)	1/17/81 (0.904)	0/12/52 (0.906)	1/5/29 (0.900)	0.536
CYP4F2*3 rs2108622 (1297 C>T)	50/43/6 (0.278)	28/32/4 (0.313)	22/11/2 (0.214)	0.054

a Data are mean values ± SD or number (%).

b Maintenance doses were determined in 89 of 99 patients.

c Data are mean values of all measured INRs.

d P-value between the INR≥4 and <4 groups.

### Study protocol

The protocol for this trial and supporting CONSORT checklist are available as supporting information; see Checklist S1 and Protocol S1. Blood sampling to measure the INR (8–13 points and 11.1 points on average) was performed at least 5 times before warfarin treatment, and at 4 days, and 1, 2 and 4 weeks after warfarin initiation with/without measurement of the plasma concentrations of warfarin enantiomers (Cp; 1–12 points and 9.2 points on average) and the fully carboxylated normal prothrombin concentration (NPT) as a biomarker of coagulation activity (4–12 points and 10.2 points on average). Thereafter, the patients were followed at least monthly for up to a maximum of 3 months. Separated plasma samples for analyses of Cp of warfarin enantiomers and NPT concentrations, and the buffy coat for DNA extraction, were stored at –80° until analysis.

### Ethics Statement

The study protocol was approved by the respective IRBs of the participating hospitals, Academia Sinica, Taiwan, and Meiji Pharmaceutical University, Japan, and written informed consent was obtained from each patient. The study protocol was registered in ClinicalTrials.gov (NCT02065388). However, as the protocol did not involve the new drug, this study was not considered as a clinical trial in Taiwan, so that the study was started without registration.

### Analyses of Cp of warfarin enantiomers and NPT concentrations in plasma

Cp of warfarin enantiomers was measured as reported previously. [22] The coagulation activity and anticoagulant effect of warfarin were assessed through measurement of the plasma NPT concentration by the carinactivase-1 method [23] and the INR value.

### Genotyping for *CYP2C9, VKORC1* and *CYP4F2*

DNA was extracted using the PUREGENE DNA purification system (Gentra Systems, MN). Allelic variants of CYP2C9*3 (rs1057910; 1075 A>C), VKORC1*2 (rs9923231; −1639 G>A) and CYP4F2*3 (rs2108622; 1297 C>T) were determined by PCR-RFLP or Taqman analysis and confirmed by direct sequencing [19], [24].

### Relationships between Dose-Cp(S), Cp(S)-NPT and NPT-INR

**Figure 2:**
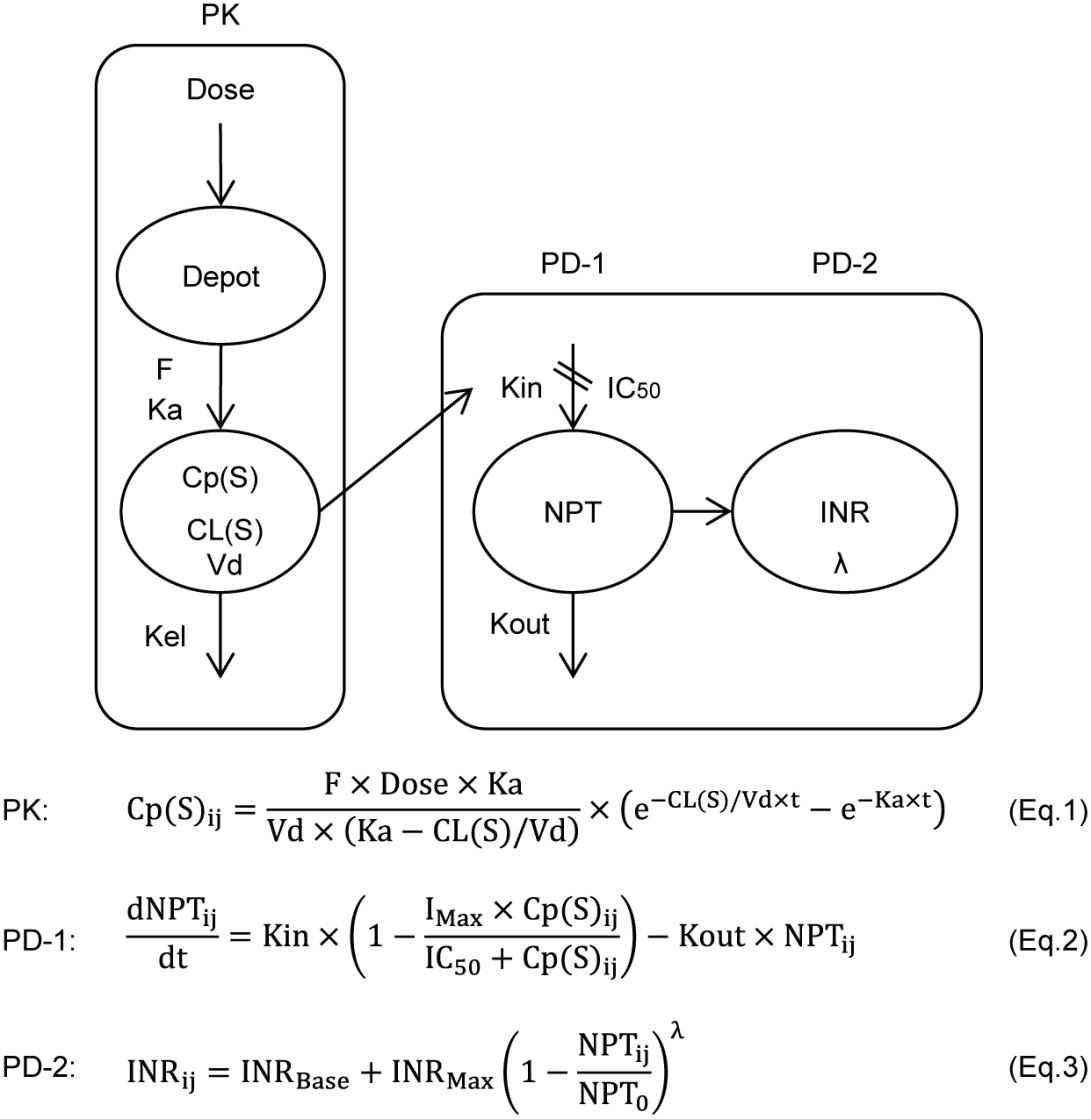
The population pharmacokinetic-pharmacodynamic (PK-PD) models of warfarin. Three models were employed to describe the time courses of the plasma concentration of *S*-warfarin, Cp(S) by the 1-compartment model (Eq.1) and normal prothrombin (NPT) by the indirect model (Eq.2) and the INR by the nonlinear model (Eq.3). Population parameters of CL(S) in PK, IC_50_ and Kout in PD-1 and λ in PD-2 were estimated.

Dose-Cp(S), Cp(S)-NPT and NPT-INR relationships (Figure 2) were analyzed using NONMEM version 7.2.0 (Icon Development Solutions, Ellicott City, MD). [25] The first-order conditional estimation (FOCE) method was used for all analyses of Dose-Cp(S), Cp(S)-NPT and NPT-INR relationships. Model selection was guided by the decrease in the objective function value (OFV). The model building was performed by the following three steps (Figure 2):

1. The time course of Cp(S) was analyzed using a 1-compartment model with the first-order absorption and elimination rate constants (Eq.1) employing the ADVAN2 and TRANS2 library routine and the population mean, the inter-individual error and covariates of CL(S) and the residual error of Cp(S) were estimated.
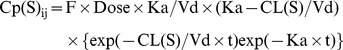
(1)where Cp(S)_ij_ represents the Cp(S) in the ith individual at the jth observation, F is the bioavailability fixed at 1.0, Ka is the absorption rate constant fixed at 2 h^−1^ and Vd is the volume of distribution of S-warfarin fixed at 13.8 L. [26] The inter-individual variability in CL(S) and the residual intra-individual variability in Cp(S) were estimated using an exponential model and an additive error model, respectively.
2. The time course of NPT concentration in response to an increase in Cp(S) after warfarin administration was described by an indirect model (Eq.2) to express the time delay between Cp(S) and NPT, in which NPT synthesis was assumed to be inhibited by the Emax model. [27] The population means and the inter-individual error of IC_50_ and Kout, covariates of IC_50_ and the residual error of NPT were analyzed employing the ADVAN6 library.
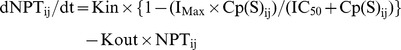
(2)where NPT_ij_ represents the NPT in the ith individual at the jth observation, Kin is expressed as Kout × NPT_0_ (baseline NPT before warfarin administration), I_Max_ is the maximum decrease in NPT concentration assumed to be 1.0 (complete inhibition of NPT synthesis), Cp(S)_ij_ is the Cp(S) in the ith individual at the jth prediction obtained using Eq.1, and IC_50_ is the Cp(S) that inhibits NPT synthesis at 50% of I_Max_. The inter-individual variabilities in IC_50_ and Kout were estimated using exponential models, and the residual intra-individual variability in NPT was estimated by an additive error model. Three patients were excluded from the NPT analysis, because NPT_0_ data were missing.
3. The time course of INR in response to a decrease in the plasma concentration of NPT after warfarin administration was described by a non-linear model based on the percentage inhibition of NPT_0_, [21] and the population mean, the inter-individual error and covariates of the exponent λ and the residual error of INR were analyzed using Eq.3: [21].
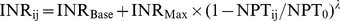
(3)where INR_ij_ represents the INR in the ith individual at the jth observation, and INR_Base_ and NPT_0_ represent the baseline INR and NPT before warfarin administration, respectively. INR_Max_ is the maximum INR increase from the baseline, which was set at 5 (the maximum INR_ij_ was fixed at 6), because the observed maximum INR_ij_ in 97.3% of the study patients was less than 6. The exponent λ accounts for the non-linear relationship between NPT inhibition and the increase in INR by warfarin. The inter-individual variability in λ and the residual intra-individual variability in INR were estimated using an exponential model and a relative error model, respectively. Since NPT_0_ data were missing in three patients, these patients were excluded from the INR analysis.

**Figure 3:**
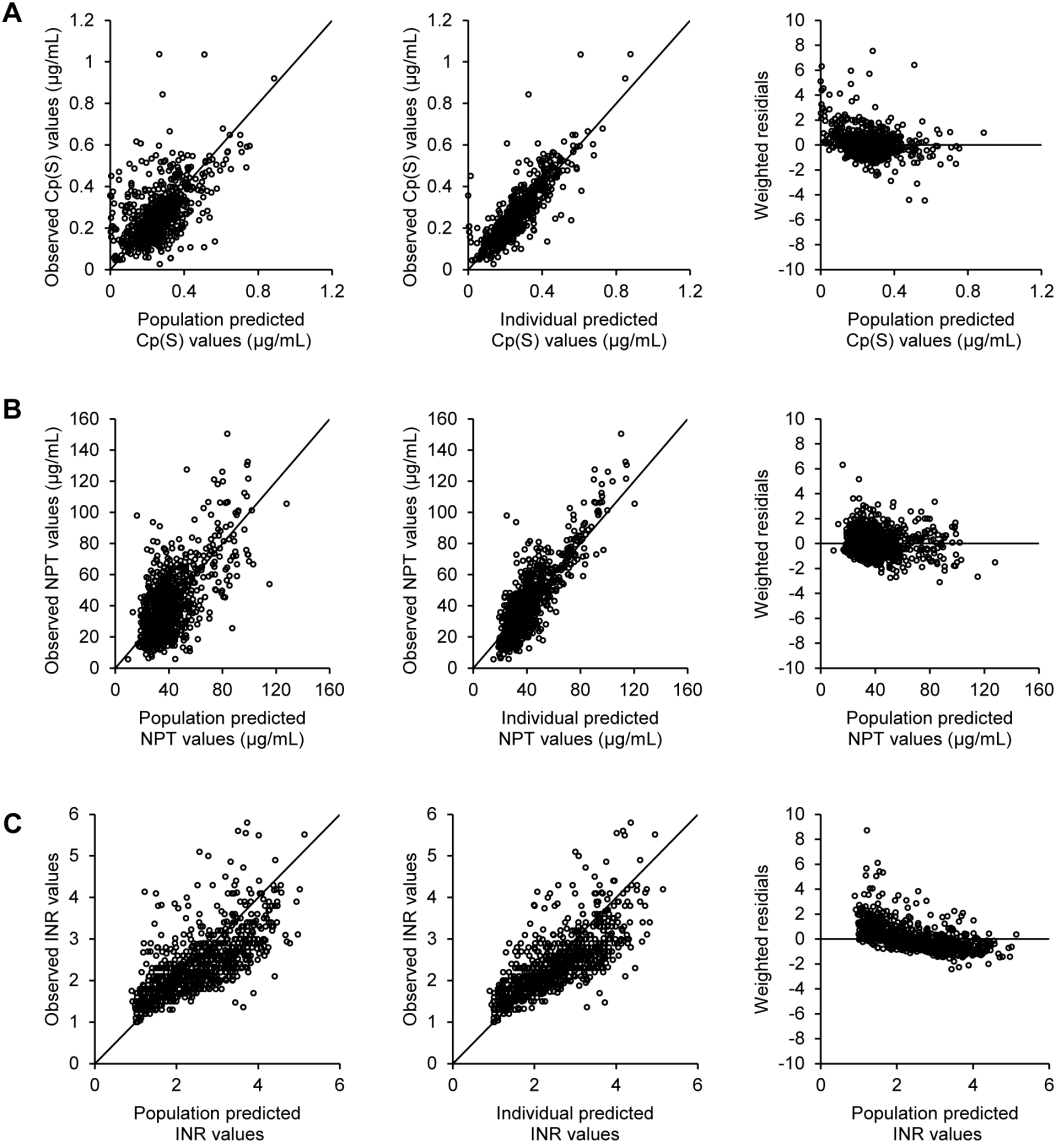
Diagnostic plots employed to evaluate population analyses. Relationships between population predictions of Cp(S) (A), NPT (B) and INR (C) and the observed values (left panel), those between individual predictions of Cp(S) (A), NPT (B) and INR (C) and the corresponding observed values (middle panel), and those between population predictions of Cp(S) (A), NPT (B) and INR (C) and weighted residuals (right panel).

The adequacy of the model predictions was checked using visual diagnostic plots of the respective population and individual predicted values of Cp(S), NPT and INR versus the corresponding observed values, and the population predicted values versus the corresponding respective weighted residuals (Figure 3). The robustness of the model was assessed using a non-parametric bootstrap procedure in which means and 95% confidence intervals (95% CI) were obtained for population parameters using 1,000 re-sampling data sets for Cp(S) and INR and 100 re-sampling data sets for NPT (Table 2). The entire procedure was performed using Wings for NONMEM (Version 720, http://wfn.sourceforge.net) [28].

**Table 2:** Summary of population PK-PD parameters for Cp(S), NPT and INR.

	Original data set		Bootstrap value	
	Mean	95% CI	Mean^a^	95% CI^b^
**PK estimates: Cp(S)**				
CL(S) (mL/h)^c^	240	220, 260	240	219, 260
Effect of BSA on CL(S)	2.14	1.12, 3.16	2.14	1.14, 3.23
Effect of CYP2C9*3 on CL(S)	0.543	0.374, 0.712	0.550	0.380, 0.723
**Inter-individual error**				
ω_CL(S)_ (%)	39.9	31.3, 48.5	38.5	27.5, 49.2
**Residual error**				
σ (µg/mL)	0.0697	0.0676, 0.0718	0.0687	0.0557, 0.0834
**PD-1 estimates: NPT**				
IC_50_ (µg/mL)^d^	0.0725	0.0631, 0.0819	0.0719	0.0629, 0.0818
Kout (1/h)	0.0136	0.0121, 0.0151	0.0138	0.0123, 0.0155
Effect of VKORC1*2 on IC_50_	2.07	1.58, 2.56	2.11	1.58, 2.65
Effect of CYP4F2*3 on IC_50_	1.30	1.07, 1.53	1.32	1.11, 1.57
**Inter-individual error**				
ω_IC50_ (%)	38.5	34.1, 42.9	37.1	31.5, 42.4
ω_Kout_ (%)	45.6	32.9, 58.3	44.0	31.1, 58.1
**Residual error**				
σ (µg/mL)	12.2	−16.6, 41.1	12.2	11.2, 13.4
**PD-2 estimates: INR**				
INR_Max_ (fixed)	5	-	5	-
λ^e^	3.48	3.30, 3.66	3.48	3.31, 3.65
Effect of NPT_0_ on λ	0.00588	0.00304, 0.00872	0.00578	0.00283, 0.00867
**Inter-individual error**				
ω_λ_ (%)	24.1	21.6, 26.7	23.4	18.1, 28.9
**Residual error**				
σ (%)	24.7	23.9, 25.5	24.6	23.1, 26.2

a Mean of 1,000 bootstrap analyses for PK and PD-2 estimates and mean of 100 bootstrap analyses for PD-1 estimates.

b The 2.5th and 97.5th values of the ranked bootstrap parameter estimates.

c CL(S) mL/h = 240×0.543^CYP2C9^*^3^×(BSA_individual_/1.74)^2.14^, where CYP2C9*3 = 0 in patients with CYP2C9*1/*1 and CYP2C9*3 = 1 in patients with CYP2C9*1/*3. BSA_median_ is 1.74 m^2^.

d IC_50 _µg/mL = 0.0725×2.07^VKORC1^*^2^×1.30^CYP4F2^*^3^, where VKORC1*2 = 0 in patients with VKORC1*2/*2, VKORC1*2 = 1 in patients with VKORC1*1/*2, CYP4F2*3 = 0 in patients with CYP4F2*1/*1 and CYP4F2*3 = 1 in patients with CYP4F2*1/*3 or CYP4F2*3/*3.

e λ = 3.48×exp{0.00588×(NPT_0individual_–119)}, where NPT_0median_ is 119 µg/mL.

Predicted time courses of Cp(S), NPT and INR in the respective patients were depicted using hourly predicted individual values.

### Multivariate analysis of predictors

The contribution of patients’ demographic predictors [i.e., age, body weight, BSA, body mass index, AST, ALT and CLcr as continuous variables, and sex, liver disease, chronic kidney disease, congestive heart failure, hypertension and history of alcohol or smoking as categorical variables] to CL(S), IC_50_ and λ, respectively, was assessed by multivariate analyses using NONMEM. In addition, the effects of CYP2C9*3 on CL(S), VKORC1*2 and CYP4F2*3 on IC_50,_ and NPT_0_ on λ were also evaluated. For respective continuous variables, e.g., the effect of BSA on CL(S), the following two models (a power model and an exponential model) were examined;
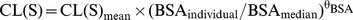
(4)

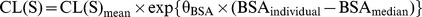
(5)


Multivariate analyses to select significant predictors of CL(S), IC_50_ and λ, respectively, involved a forward inclusion step (P<0.05 as guided by the decrease in the OFV) for the full model and a backward deletion step (P<0.01 as guided by the increase in the OFV) to build up the final model.

### Logistic regression analysis of factors associated with INR≥4

The obtained PK-PD parameters and patients’ demographic covariates, described in the multivariate analysis section above, were evaluated as independent variables contributing to INR≥4. After examining the internal correlations of each variable with CL(S) and λ, forward and backward logistic regression analyses (SPSS version 17.0) were performed. Odds ratios (ORs) and 95% CIs of significant predictors associated with INR≥4 during the initiation phase were obtained.

### Statistics

Relationships between patients’ continuous demographic data and respective CL(S), IC_50_ and λ values were examined by the Pearson correlation test. Comparisons between the median CL(S), IC_50_ and λ obtained from patients with different categorical variables, those having different *CYP2C9*, *VKORC1* and *CYP4F2* genotypes, and those with/without an INR≥4 were performed by the Mann-Whitney U test, respectively. The probability was compared using either the chi-squared test or Fisher’s exact test. The c-statistic was employed to quantify the predictability of the scheme obtained by logistic regression analyses. Data are presented as means ± SD or medians and the upper and lower quartile ranges (25th and 75th percentiles) where appropriate. A two-tailed P-value of <0.05 was considered statistically significant for all analyses. All statistical analyses were performed using SPSS version 17.0.

## Results

### Patient characteristics

The demographic characteristics of the patients are shown in Table 1. Among 99 Chinese patients, 78% received genotype-guided initiation doses of warfarin (initial dose; 4.1±1.0 mg/d for the Genotype group vs 5.0 mg/d for the Standard group, P<0.001). The maintenance doses were defined in almost 90% of the patients during follow-up periods, and the maintenance dose in Chinese patients (2.9±1.4 mg/d) was lower than those reported in Caucasian and African American patients. [2] There were significant (P<0.05) correlations between age and body weight (r = −0.358), body surface area (BSA) (r = −0.397) and CLcr (r = −0.568) (i.e., elderly patients had significantly lower body weight, BSA and CLcr). During the induction phase, 35% of the patients experienced an INR≥4 (n = 35) and were significantly older and showed higher ALT and average INR and lower maintenance dose and CLcr (P<0.05). Patients with hypertension, chronic kidney disease and atrial fibrillation showed higher rates of INR≥4 (ORs and 95% CIs; 4.7 (1.6–13.6), 5.4 (1.7–17.2), and 4.6 (1.8–11.7), respectively, P<0.01). The standard dosing group showed a higher rate of INR≥4 (13/22, 59%) than the genotype group (22/77, 29%) (OR and 95% CI; 3.6 (1.4–9.7), P<0.01). No differences were found in the initiating doses of warfarin between the INR≥4 and <4 groups.

MAFs for the three genetic variants (CYP2C9*3, CYP4F2*3 and VKORC*2) were consistent with those reported previously in Asian populations. [29] No differences in the MAFs of the three respective variants were observed between the INR≥4 and <4 groups.

### Relationships between Dose-Cp(S), Cp(S)-NPT and NPT-INR

The three models were employed sequentially to predict INR response quantitatively by warfarin, as depicted schematically in Figure 2. Overlapped means and 95% CIs for population parameters obtained from the original data set and those from the bootstrap values are shown in Table 2. A visual predictive check of the 3 models of respective Cp(S), NPT and INR was demonstrated in the diagnostic plots (Figure 3; A–C). For further confirmation of goodness of model fitting, the predicted time courses of Cp(S), NPT and INR and the observed values in the respective 34 or 35 patients with INR≥4 (NPT_0_ being missing in 1 patient) during the induction phase are shown in Figures S1–S3, respectively.

### Predictors of the PK-PD parameters and their impacts

**Figure 4:**
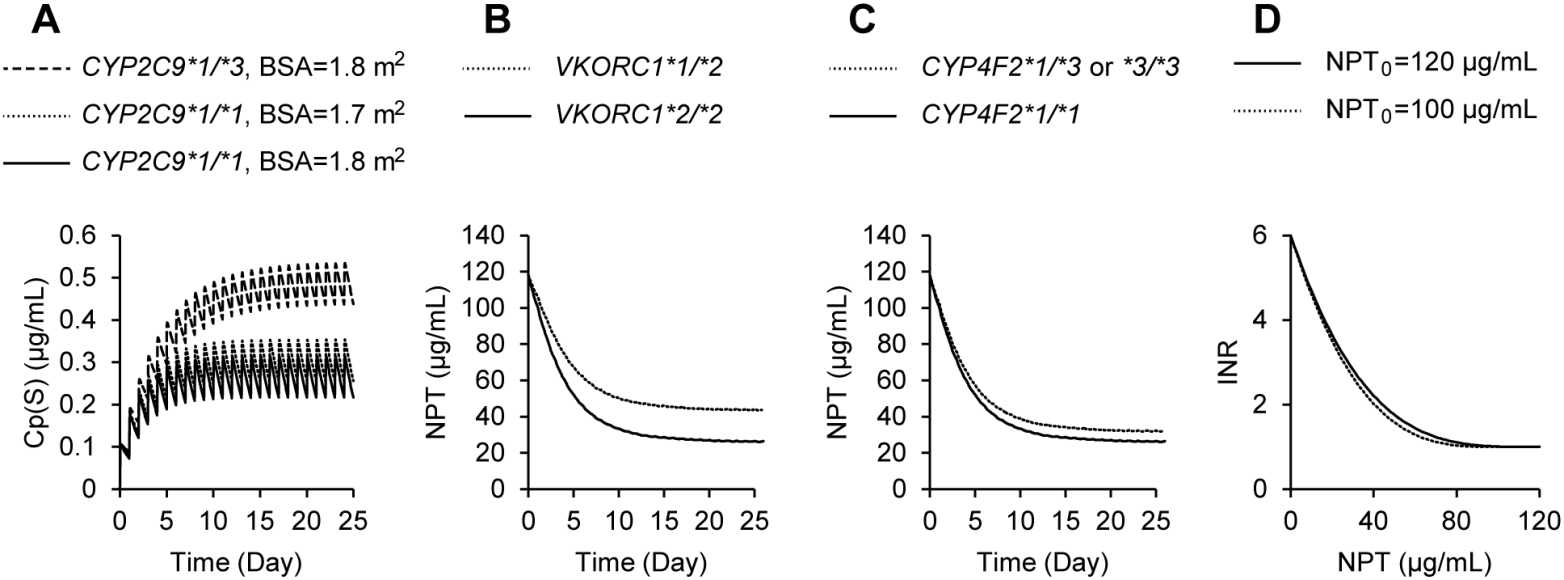
Impacts of predictors extracted from PK-PD analyses. Influences of CYP2C9*3 mutation and body surface area (BSA) on CL(S) in the time courses of Cp(S) (A), VKORC1*2 and CYP4F2*3 on IC_50_ in the time courses of NPT (B and C, respectively) and NPT_0_ on λ in the relationship between NPT and INR (D) were predicted in typical Chinese patients with a BSA of 1.77 m^2^ (165 cm and 70 kg) after administration of racemic warfarin at 3.0 mg/d.

BSA with the power model (Eq.4 in Methods) and CYP2C9*3 genotype were significant independent contributors to overall variability in CL(S), i.e., a 46% reduction in CL(S) for patients with CYP2C9*3 relative to patients with the wild type and a 13% reduction in CL(S) per −0.1 m^2^ in BSA from 1.8 m^2^ to 1.7 m^2^, which was roughly equivalent to a −10 kg reduction in body weight from 68 kg to 58 kg (height = 165 cm) (Table 2 & Figure 4). Regarding the relationship between Cp(S) and NPT, genotypes of VKORC1*2 and CYP4F2*3 were extracted as significant predictors for IC_50_, i.e., IC_50_ being 2.1 and 1.3 times larger in patients with the VKORC1*1/*2 and CYP4F2*1/*3 & *3/*3 genotypes, respectively (Table 2 & Figure 4). In the relationship between NPT and INR, baseline NPT (NPT_0_) was selected as a significant predictor for the λ value (Table 2 & Figure 4), the lower the NPT_0_, the smaller the non-linear index λ leading to a sharp increase in INR in response to the reduction of NPT after warfarin dosing. As shown in Figure 4, CYP2C9*3 mutation showed a stronger impact than that of BSA on CL(S), while mutation of VKORC1*2 had a greater influence than that of CYP4F2*3 on IC_50_ [30].

### PK-PD factors contributing to INR≥4 during the induction phase

**Figure 5:**
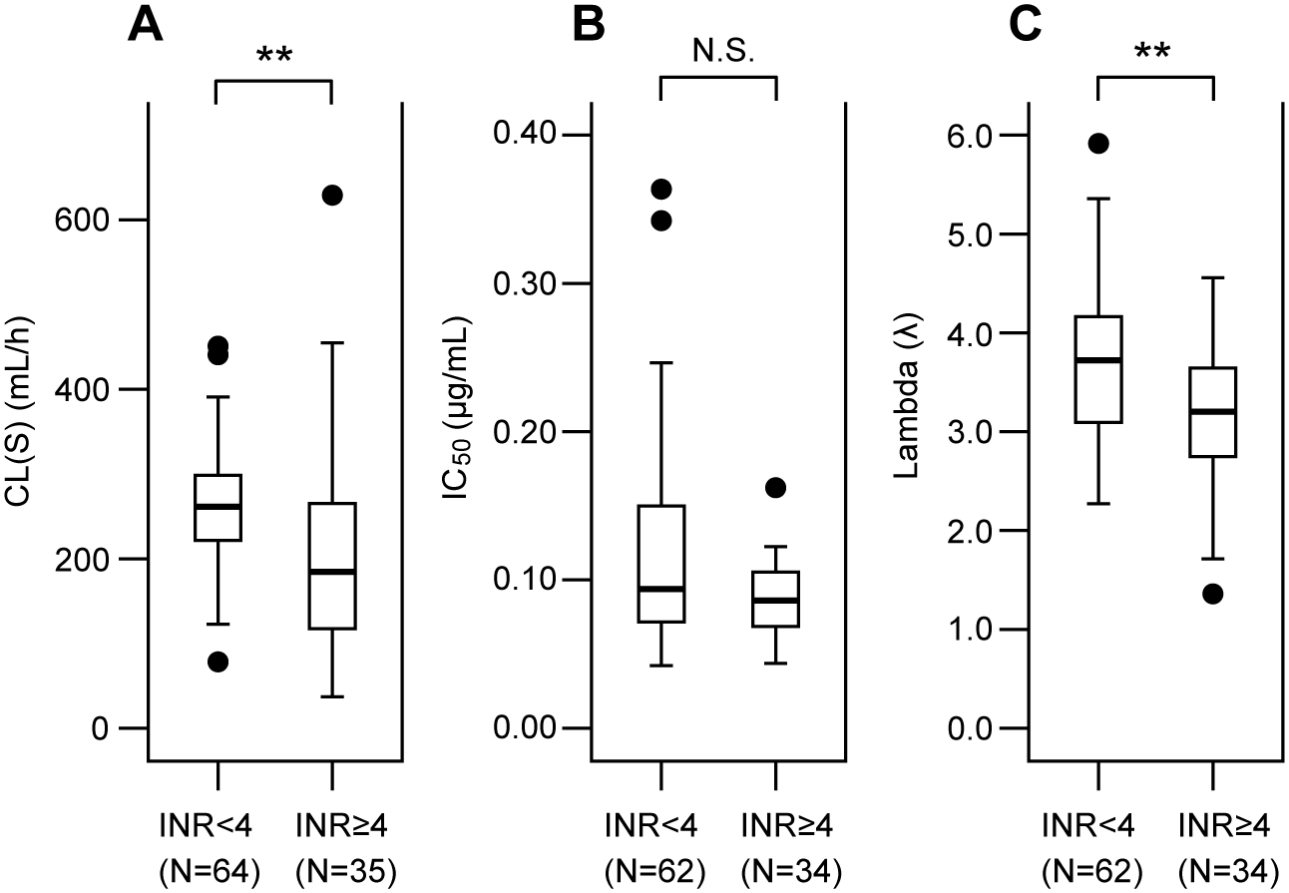
Comparisons of PK-PD parameters between the INR≥4 and <4 groups. Oral clearance of *S*-warfarin, CL(S), IC_50_ and the exponent λ were compared between the two groups of patients with an INR of ≥4 and <4 during warfarin induction treatment. Data are shown as box-and-whisker plots. The *horizontal line* indicates the median and the box covers the 25–75th percentiles. *Closed circles* (•) are outliers. **P<0.01 between the two groups.

In order to clarify the PK-PD step(s) contributing to over-anticoagulation by warfarin, individual estimates of CL(S), IC_50_ and λ obtained by population analyses were compared between the INR≥4 and <4 groups (Figure 5). This showed that patients with INR≥4 had a 30% lower CL(S) (190 mL/h vs 265 mL/h, P<0.01) and a 14% lower λ (3.2 vs 3.7, P<0.01) than those with INR<4. However, no significant difference in IC_50_ was observed between the two groups, being consistent with the similar frequencies of VKORC1*2 and CYP4F2*3 in both groups (Table 1).

### Logistic regression analysis of factors associated with INR≥4 during the induction phase

Four variables, i.e., a smaller CL(S), a greater log ALT, the presence of hypertension and absence of deep vein thrombosis, were extracted as those contributing significantly to INR≥4 (Table 3). The sensitivity, specificity and c statistic of the constructed model were 51.4%, 81.3% and 0.796, respectively.

**Table 3:** Logistic regression analysis of predictors associated with INR≥4.

Variable	β	OR (95% CI)	P-value
Constant	−2.817	0.060	0.074
*S*-warfarin clearance	−0.006	0.994 (0.989, 0.999)	0.032
log ALT	2.124	8.368 (1.191, 58.798)	0.033
Hypertension	1.318	3.735 (1.171, 11.914)	0.026
Deep vein thrombosis	−1.643	0.193 (0.049, 0.760)	0.019

**β = **regression coefficient; **OR = **Odds Ratio; **95% CI** = 95% confidence interval.

## Discussion

On the basis of a population PK-PD approach, we obtained accurate predictions for the time courses of *S*-warfarin, NPT and INR after warfarin induction (Figures 3 and S1–S3) and showed that the selected predictors of PK-PD for warfarin and their impact were consistent with previously reported data (Table 2 & Figure 4). [3], [21], [26] Most importantly, the present study is the first to demonstrate that CL(S) of warfarin, a PK determinant, and not PD factor of IC_50_, is a significant contributor to the over-anticoagulation response in Asian patients during induction therapy. This is consistent with a recent Chinese study that demonstrated an association of hemorrhagic complications with CYP2C9*3 genotype, shown to be the major PK predictor in the present study. [31] In addition to predictors of CL(S) found in the present study, e.g., the CYP2C9*3 variant and BSA, our data indicate that any factors associated with a reduction in CYP2C9 activity such as concomitant use of inhibiting drugs might potentially cause an over-anticoagulation response in Asians. Factors such as being elderly, a lower maintenance dose, higher ALT and chronic kidney disease shown in Table 1 could be potentially associated with the reduced CYP2C9 activity, thereby CL(S). These results indicate that INR should be monitored more carefully in patients receiving a lower maintenance dose of warfarin than in those receiving higher doses, as the former may be more likely to have a low CL(S).

Elderly patients are the main target population for warfarin treatment, because they have a higher risk of thrombosis, as indicated by the CHADS_2_/VASc score. [32], [33] In addition, this population has a higher bleeding risk, as shown by reported schemes/scores. [7], [8], [9] Although age was eliminated as a predictor for CL(S) and λ, because of the correlation with BSA and NPT_0_, respectively, age was significantly correlated with CL(S) (r = –0.454, P<0.01) in our population, as has been reported for whites. [26] Age-dependent reductions in the synthesis of drug-metabolizing enzymes, such as CYP2C9 and coagulation factors, as well as in liver size, may lead to lower values of CL(S) and also NPT_0_, possibly resulting in a sharp increase of the INR. [21] Overall, these results indicate that elderly patients possess multiple risk factors for over-anticoagulation with warfarin. Therefore, further studies on INR management, in terms of the time within the therapeutic range and bleeding complications, are warranted, especially for elderly patients.

The majority of our patients were given genotype-based initial doses of warfarin. The PK-PD relationship (Dose-Cp-NPT-INR) and related parameters, such as CL(S) are independent of the initiating dosing protocol, either with or without genotype information, as long as the PK of warfarin shows linearity. However, INR control and the over- anticoagulation response might be influenced by the genotype-guided dosing. This possibility might reflect the fact that the genetic algorithm was less commonly employed in the patients with INR≥4 than in those with INR<4 (62.9% vs 85.9%, P<0.01). Therefore, the contribution of CYP2C9*3 or VKORC1*2 to the over-anticoagulation response might have been underestimated in the present study, because more patients in the INR<4 group were started on warfarin doses adjusted already by their genotypes. We are currently analyzing patients data using the standard protocol to quantify the influences of CYP2C9*3 and VKORC1*2.

In this study, we were able to show that the PK process (CL(S)) is one of the main determinants of the over-anticoagulation response to warfarin in an Asian population. However, several studies have reported that *VKORC1*, the major PD determinant, has more impact than *CYP2C9* polymorphisms on early INR control and bleeding rates in white patients. [18], [34] Population differences may exist in the relationships between not only dose and Cp(S) (the PK process), but also between Cp(S) and NPT (the PD process) among whites, African American and Asian populations, as we have reported previously. [2], [35] In order to evaluate the applicability of our models constructed using data from Asian patients to white or African American patients, investigations of the PK-PD relationship for warfarin and associated predictors are essential in populations of differing ethnicity.

## Supporting Information

Figure S1 **Predicted time courses for the** ***S*** **-warfarin concentration in plasma, Cp(S).** These were depicted using individual predicted estimates of CL(S) obtained by the model analysis (Eq.1 in Method) in patients with an INR of ≥4 during the warfarin induction treatment (n = 35). *Open circles* (○) represent the observed values.
(TIF)

Figure S2 **Predicted time courses for the normal prothrombin concentration in plasma, NPT.** These were depicted using individual predicted estimates of CL(S), IC_50_ and Kout by the model analyses (Eqs.1 & 2 in Method) in patients with an INR of ≥4 during the warfarin induction treatment. As NPT_0_ data was missing in one of the patients with an INR of ≥4, 1 patient was excluded from the analyses (n = 34). *Open circles* (○) represent the observed values.
(TIF)

Figure S3 **Predicted time courses for the INR.** These were depicted using individual predicted estimates of λ obtained by the model analysis (Eq.3 in Method) in patients with an INR of ≥4 during the warfarin induction treatment. As NPT_0_ data was missing in one of the patients with an INR of ≥4, 1 patient was excluded from the analysis (n = 34). *Open circles* (○) represent the observed values.
(TIF)

Protocol S1 **Trial protocol.**
(DOC)

Checklist S2 **CONSORT Checklist.**
(DOC)
