# Supplementary figures and images for: Determinants of the Over-Anticoagulation Response during Warfarin Initiation Therapy in Asian Patients Based on Population Pharmacokinetic-Pharmacodynamic Analyses
Source: PLoS One. 2014 Aug 22;9(8):e105891. doi: 10.1371/journal.pone.0105891 (PMC4141831; doi:10.1371/journal.pone.0105891)

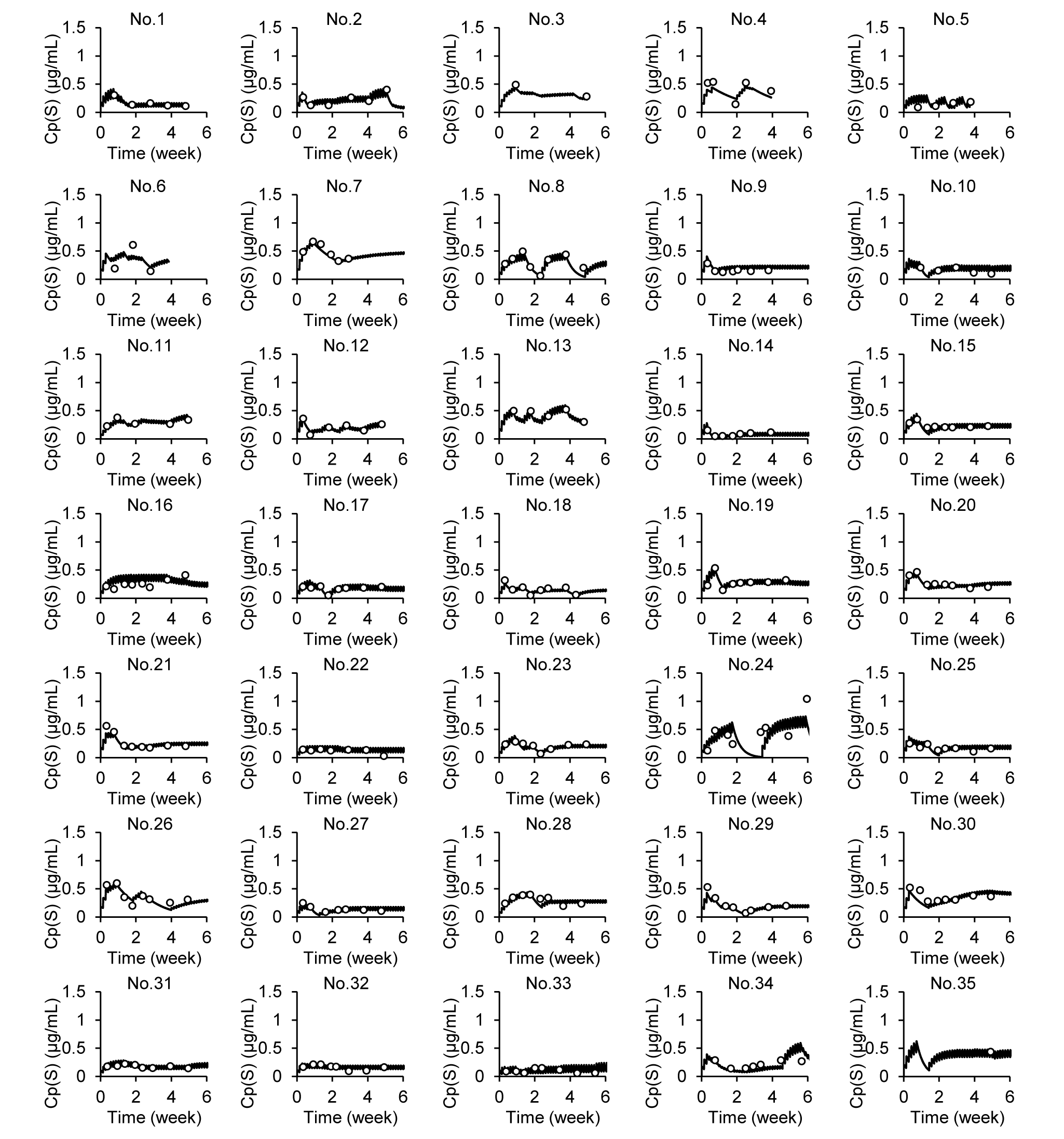

Supplement: Figure S1 — Predicted time courses for the S -warfarin concentration in plasma, Cp(S). These were depicted using individual predicted estimates of CL(S) obtained by the model analysis (Eq.1 in Method) in patients with an INR of ≥4 during the warfarin induction treatment (n = 35). Open circles (○) represent the observed values. (TIF) [file pone.0105891.s001.tif]

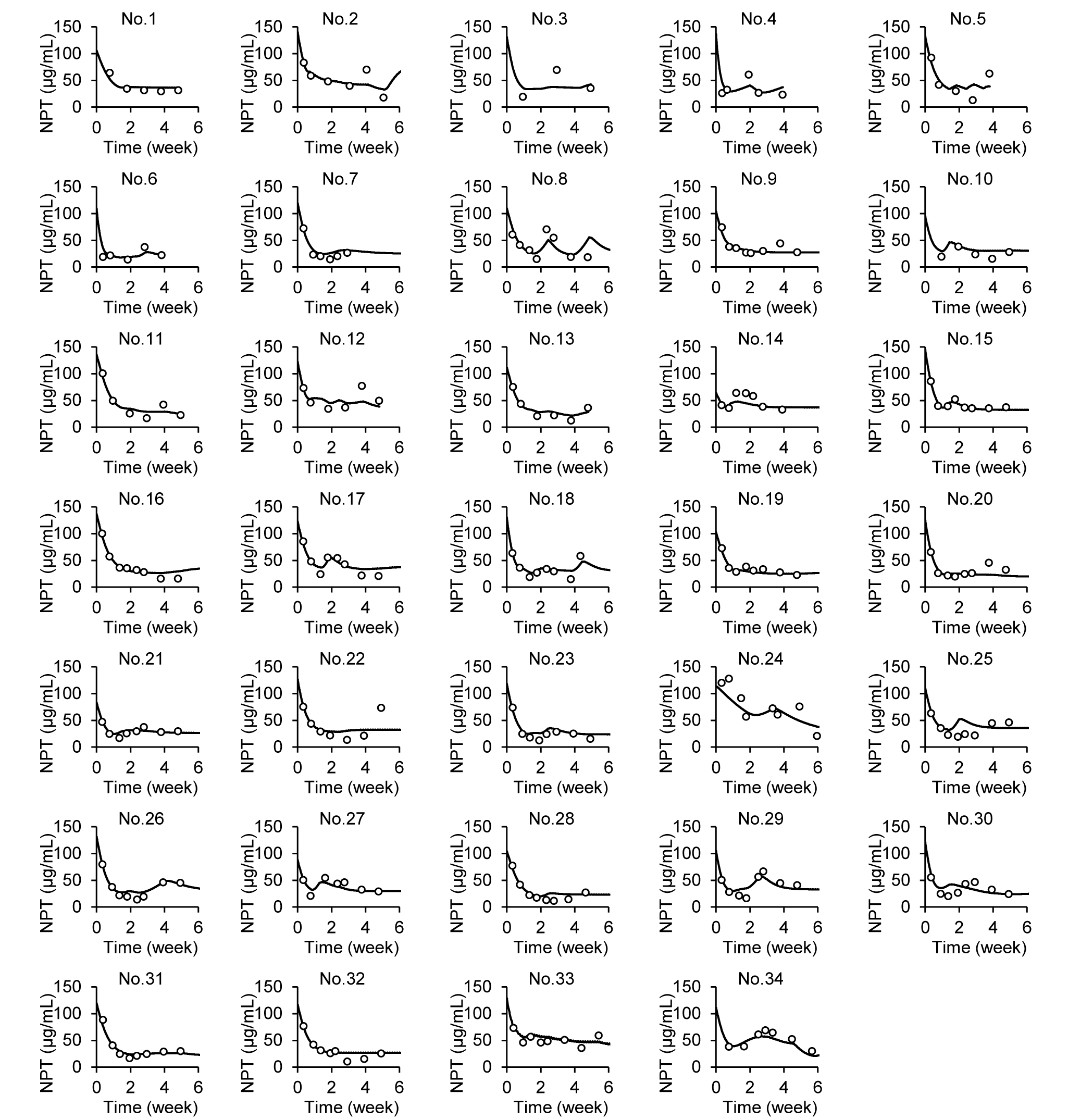

Supplement: Figure S2 — Predicted time courses for the normal prothrombin concentration in plasma, NPT. These were depicted using individual predicted estimates of CL(S), IC50 and Kout by the model analyses (Eqs.1 & 2 in Method) in patients with an INR of ≥4 during the warfarin induction treatment. As NPT0 data was missing in one of the patients with an INR of ≥4, 1 patient was excluded from the analyses (n = 34). Open circles (○) represent the observed values. (TIF) [file pone.0105891.s002.tif]

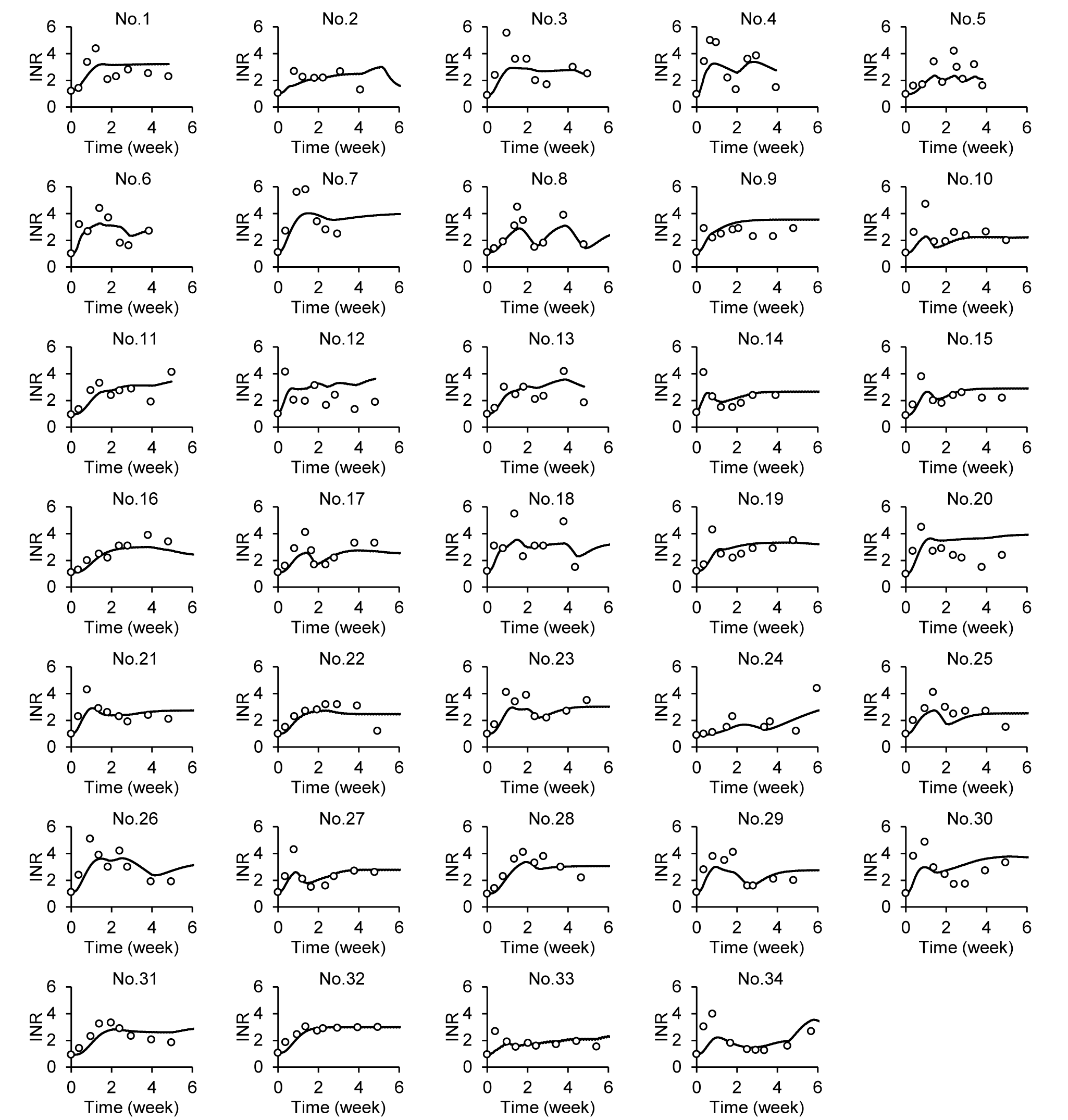

Supplement: Figure S3 — Predicted time courses for the INR. These were depicted using individual predicted estimates of λ obtained by the model analysis (Eq.3 in Method) in patients with an INR of ≥4 during the warfarin induction treatment. As NPT0 data was missing in one of the patients with an INR of ≥4, 1 patient was excluded from the analysis (n = 34). Open circles (○) represent the observed values. (TIF) [file pone.0105891.s003.tif]
