# Supplementary material for: Determinants of the Over-Anticoagulation Response during Warfarin Initiation Therapy in Asian Patients Based on Population Pharmacokinetic-Pharmacodynamic Analyses
Source: PLoS One. 2014 Aug 22;9(8):e105891. doi: 10.1371/journal.pone.0105891 (PMC4141831; doi:10.1371/journal.pone.0105891)
Supplement: Protocol S1 — Trial protocol. (DOC) [file pone.0105891.s004.doc]

Pharmacogenetic Dosing of Warfarin

A controlled Randomized Trial

Principle Investigator： 李明達

Co-Principle Investigator：

中央研究院： 鄔哲源 陳建勳

長庚紀念醫院： 溫明賢 李宗海 洪國竣

張寓智 陳俊吉 周宗川

張柏丞 周適偉

中國醫藥大學附設醫院： 張坤正 蔡輔仁 蔡崇豪

林剛旭 劉崇祥 黃偉師

國立台灣大學醫學院附設醫院： 陳錦澤 莊志明

國立成功大學附設醫院： 何中良 羅傳堯 蔡惟全

陳志弘 陳彩雲 黃溫雅

高雄醫學大學附設中和醫院： 陳英富 戴志達 許錦田

趙雅琴 林秀芬 林瑞泰

陳俊鴻 陳懷民 李碩粲

盧怡旭 林慶正 李智雄

李香君 顏學偉 蔡維中

鄭凱鴻

2009年9月21日

Protocol Signature Page

PI Signed: ______________________________

Date: ___________________

Name: 李明達

Pharmacogenetic Dosing of Warfarin

A controlled Randomized Trial

PURPOSE OF STUDY

Matching patients to the correct drug dosages that are most likely to be effective and least likely to cause harm is one of the main goals of modern therapeutics. Many drugs have narrow therapeutic ranges and any overdose can cause harm to the patients taking the drugs. Therefore, it is vital that the correct dosages are given to the patients for drugs with narrow therapeutic ranges. However, it is very difficult to predict the correct dosage. It usually takes serial trails and closely monitoring (starting from low dose) to achieve safe maintenance dose. Still, the risk still exists for extreme sensitive patients even if low doses were given first. Drugs like warfarin have a narrow therapeutic index and it can cause bleeding complications in warfarin sensitive patients. Thus, it is very difficult to determine the appropriate initial dose given to the patients. Genetic polymorphisms in the cytochrome P450 gene (CYP2C9) and the vitamin K epoxide reductase subunit 1 (VKORC1) has been shown to be responsible for warfarin sensitivity. A prospective study was conducted to test the feasibility of utilizing VKORC1 and CYP2C9 genotypes to predict warfarin dose. Data showed that the genotypes alone can predict warfarin dose for almost 70% of patients on warfarin and can reduce the time required to reach a stable and therapeutic INR. A dosing algorithm was also developed from the study which included non genetic factors to better predict the dose. Therefore, we propose a controlled, randomized trial to investigate the benefit of using the phamacogenetic dosing algorithm developed from the prospective study. In addition, dosing algorithm developed from the International Warfarin Consortium. This controlled, randomized trial will be conducted by Academia Sinica in conjunction with several major medical centers in Taiwan. It involves extensive collection of patients’ medical history, including dietary and medication and clinical samples. The recruited patients will be divided into 3 groups, two groups will be given dose as determined by the dosing algorithms and the other will receive the standard warfarin treatment. The patients groups will be blinded to the participating physicians and the patients. The patients’ genotypes will be determined using real time PCR based technology in the participating hospitals and the results will be validated at Academia Sinica. It is hoped that this trial will demonstrate the utility and benefit of pharmacogenetics as it will enables physicians to prescribe the correct warfarin dosage and will help to reduce the incidence of bleeding complications in warfarin sensitive patients. The pharmacoeconomics aspect will also be explored to assess economical benefits of pharmacogenetics.

BACKGROUND

It is well acknowledged that almost all medications display wide inter-patient variability in terms of efficacy and toxicity. These differences are sometimes greater among different ethnic groups. Some of these patients/ethnic groups can develop adverse drug reactions (ADRs), which can be fatal and this has been a major clinical problem. For many medications, these differences are caused in part by polymorphisms (mostly existed as single nucleotide polymorphism, SNP) in genes encoding drug metabolizing enzymes, drug transporters, drug targets and genes involving immune functions. Some polymorphisms will cause individuals respond to drug less effectively while others can induce adverse drug reaction or even cause death. Although many non-genetic factors, such as age, diet, nature and severity of the disease being treated, organ function, concomitant therapy and drug interactions can influence the effects of medications, however, it has been estimated that genetics can account for 20 to 95 percent of variability in drug disposition and effects (1). Unlike non-genetic factors influencing drug response, genetic factors can be inherited and generally remain stable throughout a person’s life.

ADRs can be grouped in two types, type A and type B (2). Type A ADR is the most common reaction, which can account for more than 80% of the total ADR cases. It is mostly caused by the differences in the efficiencies of drug metabolism and therefore, it is usually dose dependent. Mutations in drug metabolizing enzymes will cause the difference in drug metabolizing efficiency. Type B ADR is the rare ADR when comparing to type A. Patients who suffer type B ADR are more severe with higher morbidity and mortality. The symptoms usually involve with allergic, autoimmune, hypersensitivity and idiosyncratic reactions. It is believed that type B ADR is initiated by eliciting specific immune response against the drugs or their reactive metabolites, however, the mechanisms involved are not fully elucidated. Unlike, type A ADRS, type B ADRs are usually dose independent and unpredictable with the exception of several recent reports of association of HLA-B allele and drug hypersensitivity (3, 4).

Since not everyone responds to a drug the same way due to the existence of genetic polymorphism, it is imperative that a physician gives the correct drug/dosage to his/her patient to prevent adverse drug reaction and to maximize the effectiveness of drugs. Ideally, a physician would know what polymorphisms exist in his/her patients and use this information to give out appropriate treatment. Unfortunately, this information remains largely unavailable for many of the medications.

Under normal circumstances, drugs need to be transported to correct intracellular target sites, associated with their targets, and metabolized by the corresponding metabolizing enzymes. Therefore, any polymorphisms in drug metabolizing enzymes, drug transporters, or drug targets can cause variations in drug responsiveness. There exist some examples of how polymorphisms in certain genes can cause variability in drug response (Reviewed in 5-8).

Warfarin, a widely prescribed oral anticoagulant, is used for the prevention of thromboembolism in patients with deep vein thrombosis, atrial fibrillation, or prosthetic heart valve replacement (9-12). It has also been shown that dose response for warfarin is highly variable, both inter-individually and inter-ethnically (13-15). In addition, warfarin also has a narrow therapeutic range and thus prescribing the correct dosage is problematic. Adverse bleeding is often associated with overdose of warfarin (16-18). Much effort has been devoted to monitor the safety of this oral anticoagulant. Currently, to adjust appropriate warfarin dosage, serial determinations of blood prothrombin time using INR are needed.

Warfarin exists as a racemic mixture of (R) - and (S) - warfarin which the more potent (S)-warfarin is metabolized by CYP2C9 (19-21). Polymorphisms in this gene, such as CYP2C9*2 and CYP2C9*3, reduce the enzymatic activity and lead to reduced clearance of warfarin resulting in lower warfarin dose requirements (22). However, frequencies of these polymorphisms are low, 5% for CYP2C9*3 and non-existent for CYP2C9*2 in the Asian populations which are lower than the overall frequencies of CYP2C9 variants in the Caucasian populations (23-24). Thus, variations in CYP2C9 can not explain fully the large inter-individual and inter-ethnic differences in warfarin dose requirements. VKORC1 is the enzyme which recycles vitamin K 2,3-epoxide to reduce vitamin K required for the γ-carboxylation of vitamin K-dependent clotting factors (factor II, VII, IX, and X). Warfarin inhibits VKORC1 by reducing the regeneration of vitamin K and thus exerting its anti-coagulation effect (25-26). We reported a SNP located in the promoter region of VKORC1, -1639 G>A, which was a functional promoter polymorphism (27). The -1639 G>A SNP presented in the homozygous form (genotype AA) was found in patients who required lower doses than patients with either AG or GG genotypes in the Han-Chinese population. This was also found to be true in other Asian and Caucasian populations (29-30). The frequencies of the genotypes also correlated well with the clinical observation that Caucasian populations required higher doses than the Chinese population.

Currently, there is no accurate way to predict a patient’s warfarin dosage requirement. Prospective studies using dosing algorithms which incorporated CYP2C9 genotypes and other variables have been performed to test the validity of pharmacogenetics-based dosing, however, these algorithms could best explain approximately 40% of the variation and could not reduce the adverse outcome (31-32). An important reason may be because VKORC1 genotypes were not included. VKORC1 genotypes alone have been estimated to explain at least 15% of the variation in warfarin dosing (33-34). Using retrospective data, algorithms incorporated VKORC1 have been developed which could account for 50% of the variability in warfarin dosing (35-36). However, none of these algorithms have yet been validated prospectively in clinical trials. In this study, we tested prospectively to use CYP2C9 and VKORC1 genotypes alone to predict warfarin dose and to determine whether the pharmacogenetics-based dosing can shorten the time of achieving a stable, therapeutic warfarin dose and reduce the numbers of adverse events.

For every patient on warfarin treatment in Taiwan, the patient is usually given a low initial dose of warfarin (2.5mg/d). INR is then checked daily or every 2-3 days to monitor whether the patient reached the desired INR (INR 2-3). Depending on the INR achieved by the initial dose, warfarin dose will be adjusted. It usually takes several INR monitoring for the patient to reach the maintenance dose.

Prospective study which the warfarin dosage was prescribed based CYP2C9 and VKORC1 demonstrated that the genotype based dosing can improve the safety of warfarin treatment (37). With genotype based dosing, most patients can reach stable therapeutic INR within two weeks of treatment and the number of adverse events was also significantly reduced.

STUDY DESIGN and METHODS

The prospective study conducted (37), for the first time, demonstrated that pharmacogenetics based dosing can effectively reduce the number of adverse events and in the case of warfarin, can reduce the number of adverse events. In addition to genetic factors, nongenetic factors such as age and body surface area can also affect warfarin dosage. Therefore, dosing algorithm incorporated genetic and nongenetic factors was developed to better predict warfarin dosage using data from the prospective study. In addition, the International Warfarin Consortium also developed a dosing algorithm using data gathered from over 20 groups which contained both Caucasian and Asian ethnic groups. The study we put forward in this proposal can be described as a “pharmacogenetic clinical randomized trial”. When patients have the indications for warfarin, research nurses will explain the project to the patients and obtain their consents. Once the consents are obtained, patients’ INR will be measured and the necessary work up, such as Echo and EKG, will be performed before patients are placed on warfarin therapy. In the mean time, patients’ CYP2C9 and VKORC1 genotypes will be determined using real-time PCR based genotyping (Warfarin Dose Prediction Kit, Pharmigene) and the results will then be entered into a genotype database (iQuestion). The database contains a randomization algorithm which will automatically place the patients in one of the three groups. Patient’s blood plasma will also be analyzed for vitamin K and warfarin metabolite as this might help to explain for patients whose actual doses that deviate significantly from the predicted doses. This project will be conducted by Institute of Biomedical Sciences (IBMS), National Clinical Core (NCC) and National Genotyping Center (NGC) at Academia Sinica in conjunction with major medical centers in Taiwan (Chang Gung Memorial Hospital, China Medical University Hospital, National Taiwan University Hospital, National Cheng Kung University Hospital and Kaohsiung Medical University Hospital). The plasma analysis will be collaborated internationally with Meiji Pharmaceutical University in Tokyo, Japan. The medical centers and NCC will be responsible for patient recruitment and clinical data/sample collection. Each participant will be carefully evaluated to ensure they meet the recruitment criteria. Upon enrollment, the clinical samples (whole blood) will be transferred to the molecular diagnostic labs of participating hospitals and the National Genotyping Center, IBMS where the genomic DNA will be extracted immediately. The DNA will then genotyped to determine what polymorphisms of VKORC1 and CYP2C9 exist in these patients in the hospitals using real-time PCR based genotyping technology. The data will then be sent to the genotype database which it will assign the patients to one of the three groups. The database will then upload the assigned doses to the physicians within 1 day to allow physicians to prescribe warfarin. Genotyping will also be performed at NGC for Quality Assurance of the genotypes. The blood plasma will be frozen within 30 minutes of blood draw and stored in -80℃ and shipped to Meiji Pharmaceutical University for analysis.

Recruitment Criteria

Potential participants will be recruited if they meet the following inclusion and exclusion criteria.

*Inclusion criteria*:

In order to participate in this study, patients must meet the following.

1. Patients must give their informed consent and complete the CRF.
2. Patients must be over the age of 20.
3. Patients have clinical indications for warfarin therapy but do not have any prior warfarin treatment.

Note: When patients are placed on warfarin therapy, patients taking medications such as Amiodarone that has been shown to interfere warfarin dosing will be monitored more closely

*Exclusion criteria*:

Patients who fulfill any of the following conditions will not be eligible to participate in the study.

1. Patients who did not complete the informed consent form or the CRF.
2. Patients who are less than the age of 20.
3. Patients who had prior or is currently on warfarin treatment.
4. Patients who have hemorrhagic tendencies or hemorrhagic diseases defined as copious bleeding caused by viral or bacterial infections; cancer and hepatic dysfunction defined as GOP and GPT values three times higher than normal value
5. Patients who has Vitamin K deficiency
6. Female patients who is currently pregnant

Patients

Patient group 1: Taiwan algorithm

Patients assigned to this group will take twice the warfarin doses calculated from the prospective study algorithm.

Dose = - 0.443 + 0.798 × predict dose – 0.018 × Age + 1.4 × BSA – 0.269 × HT

Where predicted dose is based on CYP2C9 and VKORC1 genotypes (see table 1), age is in years, BSA is body surface area (m^2^) and HT indicated hypertension, =1 if the patient has hypertension, =0 if the patient does not have hypertension or status is unknown

| VKORC1 -1639 G>A | CYP2C9 | Starting Dose (mg/d) |
| --- | --- | --- |
| GG | *1/*1 | 5 |
| GG | *1/*3 | 3.75 |
| GG | *3/*3 | 3.75 |
| AG | *1/*1 | 3.75 |
| AG | *1/*3 | 2.5 |
| AG | *3/*3 | 2.5 |
| AA | *1/*1 | 2.5 |
| AA | *1/*3 | 1.25 |
| AA | *3/*3 | 1.25 |

Table 1 Predicted dose for prospective algorithm

Patient group 2: IWPC algorithm

Patients assigned to this group will use twice the warfarin doses calculated using algorithm generated by the International Warfarin Pharmacogenetic Consortium (IWPC).

Square Root of Weekly Warfarin Dose = 5.5922-

(0.2523 x Age in Decades) + (0.0089 x Height) + (0.0124 x Weight) -

(0.841 x rs9923231 C/T) – (1.690 x rs9923231 T/T) – (0.4199 x rs9923231 Missing) -

(0.5202 x CYP2C9 *1 / *2) – (0.935 x CYP2C9 *1 / *3) – (0.9789 x CYP2C9 *2 / * 2) -

(1.8313 x CYP2C9 *2 / *3) – (2.1565 x CYP2C9 *3 / *3) – (0.1486 x CYP2C9 Missing) +

(0.0821 x Asian Race) – (0.2953 x Black/African American) –

(0.1661 x Mixed/ Unspecified Race) + (1.1889 x INR decreasing drug) –

(0.6427 x Amiodarone?) – (0.3468 x Amiodarone Missing)

Where

Age in Decades = 0 for 0-10, 1 for 11-20, 2 for 21-30, etc…

Height = measured in centimeters

Weight = measured in kilograms

rs9923231 C/T = 1 if heterozygous at dbSNP entry rs9923231 else 0

rs9923231 T/T = 1 if homozygous for T at dbSNP entry rs9923231 else 0

rs9923231 Missing = 1 if no value available for dbSNP entry rs9923231 else 0

CYP2C9 *1 / *2 = 1 if heterozygous for CYP2C9 with *1 and *2 allele else 0

CYP2C9 *1 / *3 = 1 if heterozygous for CYP2C9 with *1 and *3 allele else 0

CYP2C9 *2 / *2 = if homozygous for CYP2C9 *2 allele else 0

CYP2C9 *2 / *3 = if heterozygous for CYP2C9 with *2 and *3 allele else 0

CYP2C9 *3 / *3 = if homozygous for CYP2C9 *3 allele else 0

CYP2C9 Missing = 1 if CYP2C9 genotype unknown, else 0

Asian Race = 1 if self-reported race is Asian else 0

Black/African American = 1 if self-reported race is Black/African American else 0

Mixed or Unspecified = 1 if self-reported race is mixed or unspecified else 0

INR decreasing drug = 1 if patient taking any of carbamazepine, phenytoin, rifampin, rifampicin else 0

Amiodarone? = 1 if patient taking amiodarone or amiodarone else 0

Amiodarone Missing = 1 if no data on amiodarone or amiodarone else 0

Patient group 3: Control Group

The warfarin doses for the patients in this group will not be calculated from any dosing algorithms. Instead, a standard loading dose of 5mg/day will be given.

Warfarin Dosing

The genotyping results will be uploaded to iQuestion first where the database will randomly assign patients to one of the three study groups. The assigned doses will then be sent to the physicians and given to the participants. Loading dose (1.5X of the calculated dose will be given to patients for the first three days. Patients will be educated to follow a standard precaution for warfarin therapy which is described in the appendix. Coumadin (warfarin sodium tablets) manufactured by Bristol-Myers Squibb (New York, New York, USA) will be given to all patients to ensure product consistency.

INR monitoring

After the patients receive their initial dose of warfarin, their INR will be monitor and recorded regularly. Warfarin dose will be adjusted accordingly if the INR does not fall stably between the desired ranges of 1.7-3. Once the desired INR range (INR 1.7-3) has been achieved in two consecutive visits, the maintenance dose will be recorded. Percentage of Time Patients Spend within the Therapeutic INR Range (PTTR) will also be recorded. This will serve as the primary outcome. The INR monitoring frequency is shown in table 2.

| Protocol for INR Monitoring, Stratified by Duration of Warfarin Therapy | |
| --- | --- |
| Week of Rx | INR Monitoring* |
| 1 | Mon, Thur, and day 4 of Rx |
| 2-3 | 2X/week (Mon, Thur) |
| 4-5 | 1X/week (Mon or Thur) |
| 6-9 | every other week |
| >9 | every 4 weeks |
| *INRs should also be drawn when clinically indicated (e.g. for bleeding). | |

Table 2. INR monitoring frequency.

Coagucheck portable INR (Roche) monitoring device will be used for outpatients who are not able to return to the hospitals for INR monitoring.

Dose Adjustment

Dose modifications will be made as clinically indicated and guided by a warfarin-initiation algorithm based on INR. Typically, no dose change will occur during the initial 3 days of therapy. Dose adjustment after initiation will using guideline modified from Tait el al. (1998). Warfarin dose calculated from dosing algorithms will be rounded to the next 0.25 mg. For example, an estimated dose of 3.3 mg/day would be prescribed 3.25 mg/day.

Dosing adjustment after week two will be adjusted as clinical indicated based on INR.

Dose will be adjusted if INR does not fall within the desired range of 1.7-3.

Primary outcomes :

Time to stable therapeutic INR and time spent in target INR range

Time to stable therapeutic INR and time in the target INR Range will be one of two primary endpoints because of its strong association with adverse events: patients with subtherapeutic INR values are at increased risk of thrombosis and patients with high INR values are at increased risk of hemorrhage during warfarin treatment initiation.

We will measure the time required to reach stable therapeutic INR. Decreased time to stable therapeutic INR would demonstrate the benefit of pharmacogenetic dosing.

We will examine percentage of time in the target range during days 5–30 of therapy. We will exclude days 1–4 of therapy for clinical, biological, and statistical reasons. Clinically, patients beginning warfarin therapy often are prescribed an alternative anticoagulant (e.g. a low-molecular-weight heparin) for the first 4 or 5 days of therapy, so that an optimal INR during this period is not required nor expected. Biologically, because of the long half life of some vitamin K clotting factors (eg. 30 hours for factor II) and of warfarin (average half life ~36 hours), it is difficult and probably dangerous(Harrison et al 1997) to achieve a therapeutic INR quickly. Statistically, because pharmacogenetics-based warfarin is not expected to optimize the INR during days 1–4 of therapy, including these days in the endpoint would tend to decrease our statistical power. Our primary outcome will be monitored during the initial 30 days of therapy.  We will repeat the analysis for the three-month period.

Secondary Outcomes

1. Time to Stable Warfarin Dosing

We will define stable warfarin dose as any two or more identical warfarin doses at least 2 visits apart that resulted in INR values in a slightly expanded therapeutic range (e.g. 1.8-3.2 for a target INR of 2.5).

2. Time to adverse events

Adverse events will include INR>4, Major Hemorrhage and venous thromboembolism.

Major hemorrhage is defined as major bleeds that are fatal, intracranial, require the transfusion of 2 more units of blood, or result in an acute drop of 2 gm of hemoglobin (not due to dilution). Venous thromboembolism is defined as Deep venous thrombosis or pulmonary embolism will require objective confirmation by a Doppler US, venography, pulmonary-perfusion scan, spiral CT scan, MRI, or pulmonary angiogram; a D-Dimer test will not be sufficient for a diagnosis.

1. Number of Warfarin dose adjustments in the first 30 days of therapy

Number of dose adjustments could vary by different physicians. We will also whether there is a difference in dosing patterns among the participating physicians using statistical analysis such as poisson regression model, andCochran-Armitage Chi-squared trend test.

Clinical Sample Collection

Each enrolled patients will be drawn approximately 10 ml of blood for laboratory tests and DNA extraction for the first visit. Blood will be collected in one sodium citrate-containing tube. Blood will be spun at 2500g for 10 minutes to separate the plasma player. The plasma layer and rest of the blood will be frozen in dry ice, coded and sent to National Genotyping Center, Academia Sinica for storage, and genomic DNA extraction. 5 ml of blood will also be drawn from each participant immediately after first visit following commencing of warfarin treatment and when maintenance dose is achieved for the PIVKA-II measurements. PIVKA-II (Protein Induced by Vitamin K Absence) is an abnormal des-carboxylated prothrombin, which is present in vitamin K deficiency or in patients using warfarin. By measuring PIVKA-II, it enables us to determine whether a patient has vitamin K deficiency and track a patient’s vitamin K status during warfarin treatment. It will be an indication of warfarin action allowing us to keep track of whether patients are taking warfarin regularly. Therefore, PIVKA-II will be measured before warfarin treatment and for all subsequent visits. PIVKA-II concentration will be measured using a murine monoclonal antibody available in an enzyme immunoassay kit according to manufacturer’s instructions (Asserachrom PIVKA-II; Diagnostica-Stago, Asnieres Sur Seine, France).

Genomic DNA Extraction:

Genome DNA will be extracted from the collected blood samples using PUREGENE (Gentra Systems, Minnesota, USA) purification kit according to the manufacturer’s protocol. If the blood samples are frozen, they will be first thawed in a 37℃ water bath and then undergo the extraction procedure immediately. The extracted genomic DNA will be stored in a -80℃ freezer until needed.

Genotyping and sequencing

Patient’s VKORC1 and CYP2C9 genotypes will be determined using the Warfarin Dose Prediction Kit (Pharmigene) at the participating hospitals. Genotyping results will then be validated using direct sequencing at Academia Sinica.

Note 1: There is no alteration in the treatment procedure described in this protocol. The potential participants for this project will be those who have indication for warfarin. Therefore, there will be no change of drug treatment in this project. Secondly, post treatment monitoring will not be affected either. Participants’ INR will be monitored closely. In addition, we also monitor participants’ vitamin K level to ensure that the participants do not have abnormal vitamin K concentration that could cause adverse drug reactions.

Note 2: The participants will be anonymous to protect their identity when the clinical samples and the case report forms are sent to the NGC as each participant will be code and bar-codes will be used for labeling all the materials in this study. The DNA and the plasma samples will be stored in -80C freezers in NGC where Lab Information Management System (LIMS) will be used to manage these clinical samples. The clinical information will be recorded in the iQuestion database where programs will be written to check the quality and whether errors existed in the clinical information obtained. The genotyping results will also be entered into the database. This will create a Pharamacogenetic database for warfarin which will enable us analyze both genetic and clinical data.

RISK/BENEFIT ASSESSMENT

The potential risks involved in this project are minimal. The risks for the participants in the study include the small risk of fainting or bruising and 1/1000 risk of infection at the site where the blood is drawn. The other potential risks could include the theoretical risk for discrimination towards individuals who are at-risk for a medical disorder or have a medical disorder/condition in their family. Even though the potential discrimination is highly unlikely, however, it could include barrier to insurability, employability, or remote the possibility of other unidentified adverse effects. However, extensive efforts will be made by the IBMS, NCC, and the participating medical centers to ensure confidentiality of all research participants.

The immediate direct benefits to the participants is that if the genotype is determined to be warfarin sensitive, lower dose will be given to prevent bleeding. It will also help to reduce the incidence of bleeding in their immediate family since they might inherit the same genetic variants. Finally, the greatest benefit is that the information gathered in this study will help general public to obviate the needs for multiple blood drawing for monitoring INR and reduce bleeding caused by warfarin and thus reducing or even eliminating this life-threatening side effect. Given that the risks involved with study are only minimal and the potential benefit to society is large, the risk/benefit ratio in the present study is very favorable.

SUBJECT IDENTIFICATION, RECRUITMENT, AND COMPENSATION

The potential subject for this study will be identified from the medical and surgical cardiology clinics from the participating medical centers. The study protocol will be submitted to the Institutional Review Board (IRB) of Academia Sinica and the participating medical centers for approval before any subject is recruited. The aims of the study will be explained to the participants. If they express interests in participating the study, informed consent forms (ICF), case report forms (CRF) and clinical samples (whole blood) will be collected. Those documents will be kept confidential. The subjects will be compensated with NT500 for their participation in the study.

This study aims to collect at least 120 patients for each genotype group. The exact number will be determined by how many potential subjects each medical center is able to identify and recruit. The project will be conducted from 2008/7/1 to 2011/06/30.

COSTS TO THE SUBJECT

There is no direct cost to the participant for any of the proposed research procedures.

# Reference

1. Kalow W., Tang B.K., Endrenyi I. Hypothesis: comparisons of inter- and intra-individual variations can substitute for twin studies in drug research. *Pharmacogenetics* 1998; 41:535-552.
2. Rawlins M.D. and Thompson J.W. *Mechanisms of adverse drug reactions. In Textbook of adverse drug reactions* (Davies D.M., ed.) 1991, pp18-45, Oxford University Press.
3. Chung WH, Hung SI, Hong HS, Hsih MS, Yang LC, Ho HC, Wu JW, Chen YT. *A marker for Stevens-Johnson syndrome*. Nature, 428:486 2004.
4. Hung S.I., Chung W.H., Liou L.B., Chu C.C., Lin M., Huang H.P., Lin Y.L., Lan J.L., Yang L.C., Hong H.S., Chen M.J., Lai P.C., Wu M.S., Chu C.Y., Wang K.H., Chen C.H., Fann C.S.J., Wu J.Y., and Chen Y.T. *HLA-B*5801 allele as a genetic marker for severe cutaneous adverse reactions caused by allopurinol*. Proc. Natl. Acad. Sci. USA 102(11): 4134-4139.
5. Wood A.J.J. Pharmacogenomics-Drug Disposition, Drug Targets, and side effects. *New England Journal of Medicine* 2003; 348: 538-549.
6. Evans W.E. and Johnson J.A. Pharmacogenetics: The inherited Basis for Interindividual Differences in Drug Response. *Annu. Rev. Genomics Hum. Genet.* 2001; 2: 9-39.
7. McCarthy L.C., Davies K.J., and Campbell D.A. Pharmacogenetics in diverse ethnic populations – implications for drug discovery and development. *Pharmacogenomics* 2002; 3(4); 493-506.
8. Ring H.Z. and Kroetz D.L. Candidate gene approach for pharmacogenetic studies. *Pharmacogenomics* 2002; 3(1); 47-56.
9. Hirsh, J., Dalen, J., Anderson, D.R., Poller, L., Bussey, H., Ansell, J. and Deykin, D. (2001) Oral anticoagulants: mechanism of action, clinical effectiveness, and optimal therapeutic range. *Chest,* 119, 8S-21S.

10. Laupacis, A., Albers, G., Dalen, J., Dunn, M., Feinberg, W. and Jacobson, A. (1995) Antithrombotic therapy in atrial fibrillation. *Chest,* 108, 352S-359S.

11. Stein, P.D., Alpert, J.S., Copeland, J., Dalen, J.E., Goldman, S. and Turpie, A.G. (1995) Antithrombotic therapy in patients with mechanical and biological prosthetic heart valves. *Chest,* 108, 371S-379S.

12. Hirsh, J. (1992) Antithrombotic therapy in deep vein thrombosis and pulmonary embolism. *Am. Heart J.,* 123, 1115-1122.

1. Loebstein, R., Yonath, H., Peleg, D., Almog, S., Rotenberg, M., Lubetsky, A., Roitelman, J., Harats, D., Halkin, H. and Ezra, D. (2001) Interindividual variability in sensitivity to warfarin--Nature or nurture? *Clin. Pharmacol. Ther.,* 70, 159-164.
2. Takahashi, H., Wilkinson, G.R., Caraco, Y., Muszkat, M., Kim, R.B., Kashima, T., Kimura, S. and Echizen, H. (2003) Population differences in S-warfarin metabolism between CYP2C9 genotype-matched Caucasian and Japanese patients. *Clin. Pharmacol. Ther.,* 73, 253-263.
3. Zhao, F., Loke, C., Rankin, S.C., Guo, J.Y., Lee, H.S., Wu, T.S., Tan, T., Liu, T.C., Lu, W.L., Lim, Y.T. *et al.* (2004) Novel CYP2C9 genetic variants in Asian subjects and their influence on maintenance warfarin dose. *Clin. Pharmacol. Ther.,* 76, 210-219.
4. Yu, H.C., Chan, T.Y., Critchley, J.A. and Woo, K.S. (1996) Factors determining the maintenance dose of warfarin in Chinese patients. *QJM,* 89, 127-135.
5. Xie, H.G., Kim, R.B., Wood, A.J. and Stein, C.M. (2001) Molecular basis of ethnic differences in drug disposition and response. *Annu. Rev. Pharmacol. Toxicol.,* 41, 815-850.
6. Bogousslavsky, J. and Regli, F. (1985) Anticoagulant-induced intracerebral bleeding in brain ischemia. Evaluation in 200 patients with TIAs, emboli from the heart, and progressing stroke. *Acta. Neurol. Scand.,* 71, 464-471.
7. Landefeld, C.S. and Beyth, R.J. (1993) Anticoagulant-related bleeding: clinical epidemiology, prediction, and prevention. *Am. J. Med.,* 95, 315-328.
8. Gullov, A.L., Koefoed, B.G. and Petersen, P. (1994) Bleeding Complications to Long-Term Oral Anticoagulant Therapy. *J. Thromb. Thrombolysis,* 1, 17-25.
9. Rettie, A.E., Korzekwa, K.R., Kunze, K.L., Lawrence, R.F., Eddy, A.C., Aoyama, T., Gelboin, H.V., Gonzalez, F.J. and Trager, W.F. (1992) Hydroxylation of warfarin by human cDNA-expressed cytochrome P-450: a role for P-4502C9 in the etiology of (S)-warfarin-drug interactions. *Chem. Res. Toxicol.,* 5, 54-59.
10. Kaminsky, L.S., Dunbar, D.A., Wang, P.P., Beaune, P., Larrey, D., Guengerich, F.P., Schnellmann, R.G. and Sipes, I.G. (1984) Human hepatic cytochrome P-450 composition as probed by in vitro microsomal metabolism of warfarin. *Drug Metab. Dispos.,* 12, 470-477.
11. Kaminsky, L.S., de Morais, S.M., Faletto, M.B., Dunbar, D.A. and Goldstein, J.A. (1993) Correlation of human cytochrome P4502C substrate specificities with primary structure: warfarin as a probe. *Mol. Pharmacol.,* 43, 234-239.
12. Kirchheiner, J. and Brockmoller, J. (2005) Clinical consequences of cytochrome P450 2C9 polymorphisms. *Clin. Pharmacol. Ther.,* 77, 1-16.
13. Xie, H.G., Prasad, H.C., Kim, R.B. and Stein, C.M. (2002) CYP2C9 allelic variants: ethnic distribution and functional significance. *Adv. Drug Deliv. Rev.,* 54, 1257-1270.
14. Aithal, G.P., Day, C.P., Kesteven, P.J. and Daly, A.K. (1999) Association of polymorphisms in the cytochrome P450 CYP2C9 with warfarin dose requirement and risk of bleeding complications. *Lancet,* 353, 717-719.
15. Hsiang-Yu Yuan, Jin-Jer Chen, M.T. Michael Lee, Ju-Chieh Wung, Ying-Fu Chen, Min-Ji Charng, Ming-Jen Lu, Chi-Ren Hung, Chun-Yu Wei, Chien-Hsiun Chen, Jer-Yuarn Wu, Yuan-Tsong Chen (2005) A novel functional VKORC1 promoter polymorphism is associated with inter-individual and inter-ethnic differences in warfarin sensitivity .Hum. Mol Genet., 14, 1745-1751
16. Higashi, M.K., Veenstra, D.L., Kondo, L.M., Wittkowsky, A.K., Srinouanprachanh, S.L., Farin, F.M. and Rettie, A.E. (2002) Association between CYP2C9 genetic variants and anticoagulation-related outcomes during warfarin therapy. *JAMA,* 287, 1690-1698.
17. Furuya, H., Fernandez-Salguero, P., Gregory, W., Taber, H., Steward, A., Gonzalez, F.J. and Idle, J.R. (1995) Genetic polymorphism of CYP2C9 and its effect on warfarin maintenance dose requirement in patients undergoing anticoagulation therapy. *Pharmacogenetics,* 5, 389-392.
18. Nasu, K., Kubota, T. and Ishizaki, T. (1997) Genetic analysis of CYP2C9 polymorphism in a Japanese population. *Pharmacogenetics,* 7, 405-409.
19. Bell, R.G. and Matschiner, J.T. (1972) Warfarin and the inhibition of vitamin K activity by an oxide metabolite. *Nature,* 237, 32-33.
20. Wallin, R. and Martin, L.F. (1985) Vitamin K-dependent carboxylation and vitamin K metabolism in liver. Effects of warfarin. *J. Clin. Invest.,* 76, 1879-1884.
21. Li, T., Chang, C.Y., Jin, D.Y., Lin, P.J., Khvorova, A. and Stafford, D.W. (2004) Identification of the gene for vitamin K epoxide reductase. *Nature,* 427, 541-544.
22. Rost, S., Fregin, A., Ivaskevicius, V., Conzelmann, E., Hortnagel, K., Pelz, H.J., Lappegard, K., Seifried, E., Scharrer, I., Tuddenham, E.G. *et al.* (2004) Mutations in VKORC1 cause warfarin resistance and multiple coagulation factor deficiency type 2. *Nature,* 427, 537-541.
23. Harrington, D.J., Underwood, S., Morse, C., Shearer, M.J., Tuddenham, E.G. and Mumford, A.D. (2005) Pharmacodynamic resistance to warfarin associated with a Val66Met substitution in vitamin K epoxide reductase complex subunit 1. *Thromb. Haemost.,* 93, 23-26.
24. D'Andrea, G., D'Ambrosio, R.L., Di Perna, P., Chetta, M., Santacroce, R., Brancaccio, V., Grandone, E. and Margaglione, M. (2005) A polymorphism in the VKORC1 gene is associated with an interindividual variability in the dose-anticoagulant effect of warfarin. *Blood,* 105, 645-649.

37. M-S Wen1, MTM Lee, J-J Chen, et. Al. Prospective study of warfarin dosage

requirements based on CYP2C9 and VKORC1 genotypes. Clinical Pharmacology

and Therapeutics. 2008 doi:10.1038/sj.clpt.6100453

1. Appendix: Warfarin treatment guidelines

抗凝血劑Coumadin(可邁丁)服用注意事項
1. Coumadin是一種抗凝血劑，可延長血液凝固時間，及預防血栓的產生
2. 何種情況下須使用Coumadin？
 通常是因為有血栓的產生或是有血栓的形成的危險。包括有肺栓塞、深層靜脈栓塞、

風濕性心臟病、人工瓣膜置換等
3. 服用的時間長短則依每個病患而不同，有的甚至須終身服用，如沒有醫師開立醫囑，

不可擅自停藥或增量
4. 而Coumadin的使用劑量也因病患而有所不同，醫師會依據一種血液測試，即凝血酵

素原時間(Prothrombin Time)來監測，並調整適合的劑量，這種血液測試在治療初

期的監測較頻繁，一旦劑量調整好後，次數便可減少
5. 換金屬瓣膜之病人，需終生服用，以預防血栓形成
6. 服用前請仔細辨認，並按照醫師指示服用，避免因服用劑量過少造成用藥無效，或

劑量過多導致出血等合併症
7. 如欲拔牙，肌肉注射或行其他手術，應請教心臟專科醫師，以免造成流血不止
8. 維他命K會拮抗可邁丁(Coumadin)的功能，所以富含維他命K之綠色蔬菜如：菠菜、

甘藍菜及綠色花椰菜應避免大量攝取
9. 木耳及菇類食物之作用剛好相反，會增可邁丁(Coumadin)之作用最好不要食用
10.儘量保持您固定飲食習慣，避免因您的飲食型態改變太大而影響藥物的作用
11.有許多藥物會影響可邁丁(Coumadin)作用，如阿斯匹靈(Aspirin)，百服寧

(Bioform)或非類固醇類的消炎藥等；另外應避免同時服用中藥，以免影響藥效
12.如有下列情形，例如：皮膚容易瘀青、小便變紅、齒齦易出血、咳血、流鼻血、

解血便等現象，請先行停止服用可邁丁(Coumadin)並立即請教您的專科醫師或至

心臟科門診就診；若出血嚴重，則應到急診室求診
13.如果因為有其他任何不適，去別家醫院或診所就醫，請告知醫師，有服用抗凝血劑
14.服用Coumadin的患者，仍然可從事一些安全的運動，如慢跑、游泳，這些運動也

可以幫助血液循環，但會有身體衝撞的劇烈運動，則應該避免以減少受傷機會
15.需拔牙時應先停用抗凝血劑五天後再行拔牙，止血後須按照專科醫師指示繼續服用

抗凝血劑

16.影響可邁丁(Coumadin)作用的草本藥食

抑制凝血(出血危險性增加)：銀杏、當歸、丹参、木耳

加強凝血(血管栓塞可能性提高)：人參

| 100克食物 維他命K含量之參考 | | | 每100克食物 維他命K含量之參考 | |
| --- | --- | --- | --- | --- |
| 低 (<50μg) | | 中度(50-100μg) | 高 (100-200μg) | 很高(>200μg) |
| 蘋果 | 青蘋果 | 馬鈴薯 | 九層塔 | 綠花菜 |
| 酪梨 | 蘆筍 | 米(榖類) | 硬花甘藍 | 碗豆/扁豆 |
| 胡蘿蔔 | 高麗菜 | 蕃茄 | 菜籽油 | 萵苣 |
| 芹菜 | 花椰菜(白) | 玉米 | 蝦夷蔥 | 肝臟 |
| 麥片 | 蛋黃醬 |  | 洋蔥/青蔥 | 荷蘭芹菜 |
| 咖啡 | 堅果(開心果) |  | 帶皮黃瓜 | 菠菜 |
| 蛋 | 南瓜 |  | 芥菜 | 紅茶 |
| 水果 |  |  | 大豆油 | 綠茶 |
